# Contributions of the Primary Sensorimotor Cortex and Posterior Parietal Cortex to Motor Learning and Transfer

**DOI:** 10.3390/brainsci14121184

**Published:** 2024-11-26

**Authors:** Chenyu Wang, Yinghua Yu, Jiajia Yang

**Affiliations:** Graduate School of Interdisciplinary Science and Engineering in Health Systems, Okayama University, 3-1-1 Tsushima-Naka, Kita-ku, Okayama 700-8530, Japan; pv3g8zph@s.okayama-u.ac.jp (C.W.); yinghua.yyh@gmail.com (Y.Y.)

**Keywords:** fMRI, motor learning and transfer, primary sensorimotor cortex, posterior parietal cortex

## Abstract

Background: Transferring learned manipulations to new manipulation tasks has enabled humans to realize thousands of dexterous object manipulations in daily life. Two-digit grasp and three-digit grasp manipulations require different fingertip forces, and our brain can switch grasp types to ensure good performance according to motor memory. We hypothesized that several brain areas contribute to the execution of the new type of motor according to the motor memory. However, the motor memory mechanisms during this transfer period are still unclear. In the present functional magnetic resonance imaging (fMRI) study, we aimed to investigate the cortical mechanisms involved in motor memory during the transfer phase of learned manipulation tasks. Methods: Using a custom-built T-shaped object with an adjustable weight distribution, the participants performed grasp and lift manipulation tasks under different conditions to simulate the learning and transfer phases. The learning phase consisted of four grasp-and-lift repetitions with one motor type, followed by a transfer phase with four repetitions involving different motors (adding or removing a digit). Results: By comparing brain activity in the learning and transfer phases, we identified three regions (the superior frontal gyrus, supramarginal gyrus, and postcentral gyrus) associated with motor memory during the transfer of learned manipulations. Conclusions: Our findings improve the understanding of the role of the posterior parietal cortex in motor memory, highlighting how sensory information from memory and real-time input is integrated to generate novel motor control signals that guide the precise reapplication of control strategies. Furthermore, we believe that these areas contribute to motor learning from motor memory and may serve as key regions of interest for investigating neurodegenerative diseases.

## 1. Introduction

Dexterous manipulation of various objects by humans depends on learning motors and transferring this learned information from memories of similar motors. Functional magnetic resonance imaging (fMRI) has been proven to be a powerful tool to explore the brain mechanisms underlying motor learning from various stages [1,2,3,4]. These stages include a fast learning stage at first manipulation, stabilization of manipulation memories with following practice, and transfer to new types. In this study, we focused on the motor learning and transfer mechanism in grasping and lifting objects, specifically when participants switch grasp types (adding or removing digits to grasp) [5,6]. As two-digit grasping [5,6,7] and three-digit grasping [8] motors require different fingertip forces and hand muscle forces, the transfer period for adding or removing a digit depends on new motor control strategies. Although such motor control strategies have been validated through changes in grasp forces at the behavioral level [5], the brain mechanisms underlying motor control during the transfer learning processes of grasp-and-lift manipulations remain unknown.

Dynamic sensorimotor interaction processing mechanisms are essential in motor control [9,10,11,12]. Without knowledge of the weight distribution of the object, the human brain dynamically interacts with the environment to recognize changes and determine appropriate motor outcomes [13,14,15]. The primary somatosensory cortex (S1) and motor cortex (M1) have been shown to be the first-level brain areas involved in sensorimotor interactions. The secondary somatosensory cortex (S2), posterior parietal cortex (PPC), prefrontal cortex (PFC), and premotor cortex (PMC), which are considered higher-level areas, are also involved in both haptic and tactile object processing [16,17,18]. Owing to uncertain estimates of the object’s weight and distribution, when grasping and lifting objects, online sensory feedback is needed to determine the next motor and appropriately adjust the force applied [19]. We aimed to investigate whether these sensorimotor and high-level areas play a role in manipulating objects.

Through repeated motors, our brain learns how to apply not only net vertical forces but also torques to control the manipulation [20,21,22,23]. During the grasping and lifting process, sensory input from the fingertips, combined with motor performance, results in the formation of memories that enable stable manipulation of the objects in subsequent trials [24,25,26,27]. With the formation of memories, we hypothesized that during repeated manipulations with the same object, sensorimotor interactions would be reduced, which might be reflected by fewer brain areas being involved in the motor control of a learned manipulation.

If the control of the manipulation relies on the motor memory, and the same force used in the memory is applied, switching the grasp type, such as by adding or removing digits, would compromise the behavioral outcome during lifting. However, behavioral evidence [5,6,28] has shown that such skilled manipulations could still be performed accurately when executing new types of manipulations. Brain areas involved in cortical motor prediction or motor planning mechanisms may be responsible for the transfer of learned manipulations to novel tasks. Such motor transfer learning occurs when subjects perform a manipulation using a new type of motor, which can be defined as the transfer phase. According to a Bayesian perspective of predictive processing, the brain enables actions to be performed by integrating prior information with current feedback and then making and/or using predictions [29,30]. During the transfer period of a learned manipulation, S1 and M1 may communicate with higher-level areas, such as the PFC and PPC, to generate new motor control signals and facilitate novel sensorimotor interactions, along with other higher-order processes [31,32,33]. However, how the interactions between high-level areas and S1 and M1 contribute to motor control during the transfer of learned manipulations to new types of manipulations remains unknown.

In the present fMRI study, we aimed to investigate the cortical mechanisms involved in motor control during the learning and transfer phases of grasp and lift manipulations. A T-shaped object with an adjustable weight distribution was used to create different learning and transfer scenarios. The object consisted of a vertical stick at the top, designed for participants to grasp, and a horizontal frame with three compartments to hold weights for adjusting the distribution. The same object with different weight distributions was considered a new object, and participants experienced both learning and transfer periods while grasping and lifting the object eight times in the fMRI experiment. The first four grasp-and-lift motors were defined as the learning period, followed by the transfer period, in which participants changed their grasp by either adding or removing a digit. By comparing brain activity in the initial learning periods and the transfer periods, we aimed to investigate cortical activation associated with changes in motor memory throughout the manipulation learning process, particularly during the transfer phase.

## 2. Methods

### 2.1. Participants

Eighteen healthy right-handed participants (all males, aged 22–24 years, with a mean age of 22.1 years) were included in the fMRI experiments. Considering that male participants typically possess sufficient physical strength to complete the entire fMRI experiment, we selected male participants for our fMRI experiments. However, we acknowledge the limitations of gender diversity. None of the participants reported abnormal tactile sensations, a history of major medical or neurological illness, or a history of alcohol dependence. All the participants provided written informed consent. The experimental protocol was approved by the local Medical Ethics Committee at Okayama University Hospital.

### 2.2. Grasp and Lift Manipulation Experiments

#### 2.2.1. Apparatus

**Figure 1:**
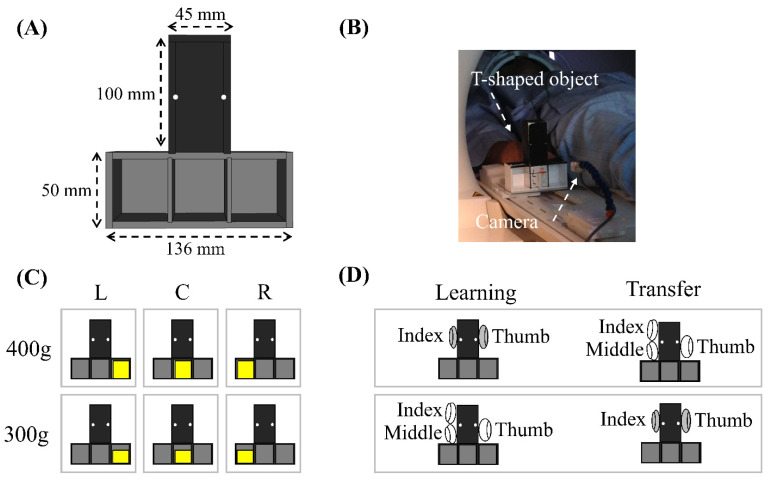
(**A**) Configuration of the T-shaped object. (**B**) A photo of the fMRI experimental setup. (**C**) Diagram of the 6 kinds of weight distribution of the T-shaped object. A mass (400 or 300 g) was inserted in either the left (L), right (R), or center (C) compartment at the bottom of the device to change the weight distribution of the object. (**D**) Diagram of transferring the learned manipulation by adding middle finger (2d to 3d) and removing middle finger (3d to 2d).

In the experiment, a T-shaped object whose weight distribution could be manually adjusted was utilized. The weight distribution was adjusted by placing a mass in various locations within the T-shaped frame, as shown in Figure 1. The object consisted of a vertical stick at the top and a horizontal frame at the bottom, both with thicknesses of 40 mm. The vertical stick, measuring 100 mm × 45 mm and centrally positioned, was designed to be grasped by the subject. Referring to the mass used for adjusting weight distribution in a similar grasp and lift task [34], we selected the 300 g and 400 g masses for our experiment. The bottom part of the frame contained three compartments, and a mass (300 g or 400 g) could be placed in one of the compartments to adjust the weight distribution of the object. By placing the mass in one of the compartments, the overall weight distribution could be shifted to the left, right, or center.

#### 2.2.2. Task Procedure

During the fMRI experiment, participants lay on the scanner bed with their right arm and hand positioned to grasp the object. The participants were instructed to watch a projection screen and perform the motor according to the on-screen instructions. The projector was placed 4.5 m away from the scanner, and the instructions were displayed on an experimental control PC triggered by the MRI scanner.

**Figure 2:**
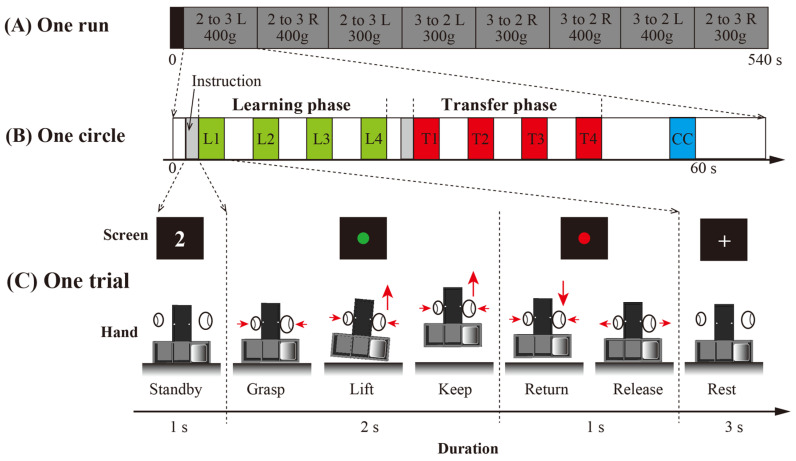
(**A**) Diagram of the experimental paradigm. Each subject participated in five 540 s runs. Each run includes one 12 s baseline period and eight 66 s parts. (**B**) Diagram of one circle. Each circle included two 1 s instruction periods, four 3 s learning trials (L1–L4), four 3 sec transfer trials (T1–T4), and one 3 s center mass control trial (CC). (**C**) Diagram of one trial. The participants fixated on the visual cues on the screen. Before each learning and transfer phase, “2” or “3” was presented on the screen to ask the participants to perform the following trials using 2 or 3 digits. Then, the participants were asked to grasp the device when the green stimulus was presented and release it when the red visual stimulus was presented.

A diagram of the experimental paradigm is shown in Figure 2. Each participant completed five runs in a single fMRI experiment. Each run consisted of eight randomly arranged circles: two weights (300 g or 400 g) × two types of weight distributions (left or right compartment) × two types of transferring the learned manipulation (adding or removing a digit). Before the start of each circle, the experimenter manually placed one of the two weights into either the left or right compartment of the T-shaped object, creating a new weight distribution. This alteration allowed every circle to be treated as a new object manipulation task. In other words, participants underwent the complete learning and transfer phases of the grasp-and-lift manipulation under the same weight distribution condition in each circle.

Each circle consisted of a learning period, a transfer period, and a control period. Before the learning and transfer periods, the participants were informed whether they would use 2 digits (thumb and index finger) or 3 digits (thumb, index finger, and middle finger) to grasp and lift the object. In the control period, participants would grasp the object with the same grasp type as the one used in the transfer period. The learning and transfer periods involved four consecutive grasp and lift manipulation trials, whereas the control period included only one manipulation trial. Specifically, the first four trials (L1, L2, L3, and L4) were defined as learning period trials, whereas the following four trials (T1, T2, T3, and T4) were defined as transfer period trials. In the control period, a center mass clear trial (CC) was introduced between the transfer trials and the next set of learning trials to eliminate carryover effects. In each trial, when a green circle appeared on the screen, the participants were instructed to grasp and lift the object while minimizing any rolling motor. Two seconds later, when a red circle appeared, the participants were instructed to return the object to its starting position.

### 2.3. fMRI Image Acquisition

MRI datasets were collected with a Siemens MAGNETOM Verio 3T MRI scanner (Siemens, Erlangen, Germany). An echo planar imaging (EPI) sequence with the following parameters was chosen to acquire the functional images: repetition time (TR) = 3000 ms, echo time (TE) = 30 ms, flip angle = 90°, matrix = 82 × 82, number of axial slices = 40, in-plane field of view = 192 × 192 mm^2^, and slice thickness = 3.0 mm (whole-brain coverage). After the EPI sequence, a T1-weighted anatomical image with a voxel size of 0.977 × 0.977 × 1.0 mm^3^ (matrix 256 × 256 × 224) was acquired.

### 2.4. fMRI Data Analysis

We used the statistical parametric mapping (SPM12) package [35] with Matlab R2018b (The Math Works Inc., Natick, MA, USA) to process and analyze the fMRI data. The first four scan volumes of each run were discarded because of unsteady magnetization. The EPI datasets of each subject were preprocessed following the same steps.

1. For each run, the first 4 volumes were removed for magnetization equilibration. 2. Slice-timing correction was conducted to adjust for differences in slice acquisition times. 3. Functional images from each run were realigned to the mean image. 4. The EPI datasets of each subject were aligned to the anatomical T1 space (Montreal Neurological Institute (MNI) space). 5. The normalized functional images were filtered with a Gaussian kernel with an 8 mm full width at half maximum (FWHM) along the x-, y-, and z-axes. 6. The parameters from this normalization process were then applied to the functional images, which were resampled to a final resolution of 2 × 2 × 2 mm^3^.

#### 2.4.1. Initial Individual Analysis

A general linear model was applied to the fMRI data for each subject. The blood-oxygen-level-dependent (BOLD) signals for all conditions were modeled with boxcar functions convolved with the canonical hemodynamic response function (HRF). The design matrix for each subject included nine regressors, representing the four grasp and lift manipulations during the learning and transfer periods and the one motor in the control period. Motor-related artifacts were controlled by incorporating six motion parameters (three translations and three rotations) from the rigid-body realignment process into the model. The serial autocorrelation was estimated from the pooled active voxels via a first-order autoregressive model and the restricted maximum likelihood (ReML) procedure, which was then used to whiten the data [36]. Estimates were assessed with linear contrasts comparing the nine movement trials to the baseline for each subject, and the resulting contrast images were used for group analysis.

#### 2.4.2. Group Analysis

Initially, one-sample *t*-tests were conducted at the group level for the eight regressors (L1 to L4 and T1 to T4) to identify brain activity during the grasp and lift tasks across the different periods. The significance threshold was set at *p* < 0.05, which was corrected for multiple comparisons via the familywise error (FWE) correction across the whole brain, and the cluster extent threshold was set at k > 20 voxels. The activation maps for the learning and transfer periods were then generated and overlapped, with the results shown in MNI space.

To investigate brain activity related to motor prediction, we compared brain activity during T1 with that during L4. From this comparison, we extracted time series signals from 8 mm diameter spherical regions of interest (ROIs) centered on the peak coordinates of areas showing stronger activation in T1 than in L4. To compare the % signal change in T1 and L4 in each ROI, we performed paired two-tailed *t*-tests using the R package in RStudio [37]. The Benjamini–Hochberg correction (False Discovery Rate; FDR) was applied for multiple testing.

## 3. Results

### 3.1. Whole-Brain Neural Activity for Grasp and Lift Manipulations During the Learning Period Compared with the Baseline

**Figure 3:**
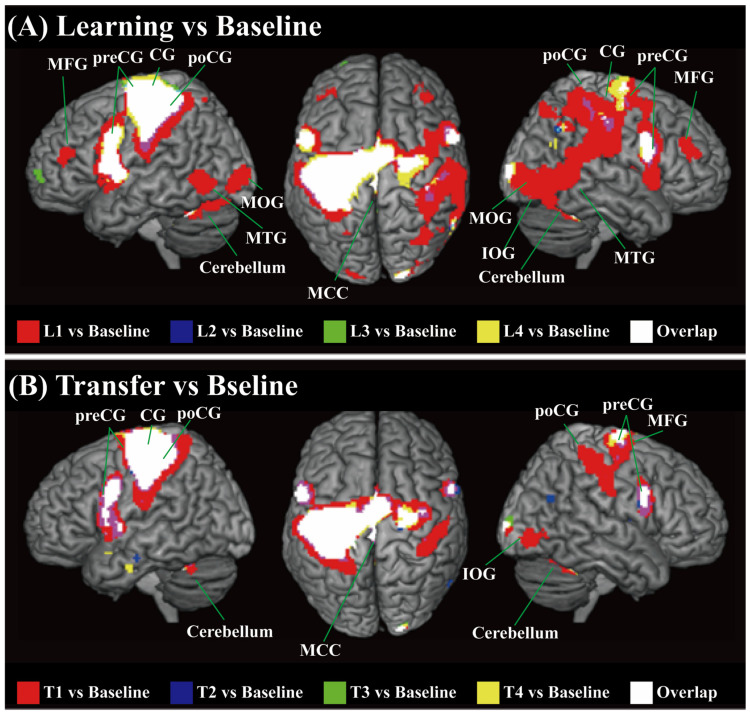
Cortical activation patterns in (**A**) the learning period vs. the baseline period and (**B**) the transfer period vs. the baseline period were superimposed on surface-rendered high-resolution MR images unrelated to the participants in the present study, viewed from the left, top, and right of the brain. Regions in white were activated during all tasks (overlap). The significance threshold was set at *p* < 0.05, which was corrected for multiple comparisons (FWE) across the whole brain. SFG: superior frontal gyrus; MFG: middle frontal gyrus; SMG: supramarginal gyrus; CG: central sulcus; PoCG: postcentral gyrus; preCG: precentral gyrus; MCC: middle cingulate cortex; MOG: middle occipital gyrus; IOG: inferior occipital gyrus; MTG: middle temporal gyrus.

As shown in Figure 3A, we confirmed that the four manipulations in the learning period activated a wide range of brain areas, and the regions activated during these four learning trials showed high overlap, as indicated by the white colors in the figure. The brain regions that were consistently activated across the four learning trials included the contralateral precentral gyrus (preCG), central sulcus (CG), postcentral gyrus (poCG), middle frontal gyrus (MFG), middle cingulate cortex (MCC), and bilateral cerebellum. In addition to the contralateral S1 and M1 regions, we detected activations in the contralateral supplementary motor area (SMA) and bilateral dorsal and ventral premotor cortex (dPMC and vPMC, respectively). Notably, more brain areas were activated during the first learning trial, including the ipsilateral preCG, CG, poCG, bilateral MFG, middle occipital gyrus (MOG), inferior occipital gyrus (IOG), and middle temporal gyrus (MTG).

### 3.2. Whole-Brain Neural Activity for Grasp and Lift Manipulations During the Transfer Period Compared with the Baseline

As shown in Figure 3B, we observed that the four motors with different grasp types in the transfer period activated a wide range of brain areas, and the overlap in the regions activated during these four motors during the transfer period was nearly identical to the overlapping activation areas in the learning period, including the contralateral S1, M1 and SMA, bilateral dPMC, vPMC, and cerebellum. Additionally, more regions, including the ipsilateral S1 region and the IOG, were activated during the first manipulation than during the subsequent three manipulations.

### 3.3. Brain Activities Associated with Transferred Motor Learning

**Figure 4:**
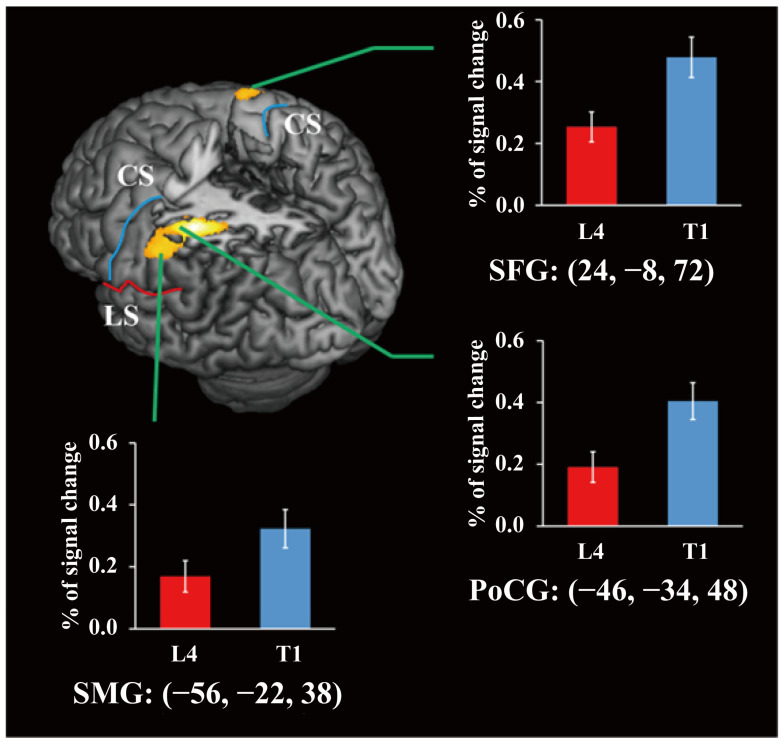
The brain regions more strongly activated during T1 (the first trial in the transfer period) than during L4 (the last trial in the learning period) were superimposed on surface-rendered high-resolution MR images unrelated to the participants in the present study. The significance threshold was set at *p* < 0.05, which was corrected for multiple comparisons (FWE) across the whole brain, and the extended threshold was set at k > 20 voxels. The colored bar graphs indicate the task-related activation (% signal change) during L4 and T1 in 3 ROIs (8 mm diameter sphere). The centers of the spheres were the coordinates for the peak activation. The error bars indicate the standard error of the mean. CS (blue line): central sulcus; LS (red line): lateral sulcus; SFG: superior frontal gyrus; SMG: supramarginal gyrus; PoCG: postcentral gyrus.

To observe the brain regions involved in the transfer of the grasp type for a given manipulation, we compared the brain regions activated during the grasp and lift manipulations of the first trial in the transfer period with those activated during the last trial in the learning period. As shown in Figure 4, three areas, including the superior frontal gyrus (SFG), supramarginal gyrus (SMG), and PoCG, were activated more strongly in the transfer period than in the learning period under the threshold; *p* < 0.05 (FWE-corrected). Furthermore, the time series analysis of the ROI revealed stronger task-related activation (% signal change) in T1 than in L4 (SMG: t(17) = 5.26, *p* < 0.001, Cohen’s d = 1.24; SFG: t(17) = 4.10, *p* < 0.001, Cohen’s d = 0.97; PoCG: t(17) = 6.02, *p* < 0.001, Cohen’s d = 1.42. FDR corrected).

## 4. Discussion

In this study, we investigated brain activity during grasp and lift manipulations in both the learning and transfer periods of an fMRI experiment. By adjusting the weight distribution, the participants were uncertain about the weight distribution of the object in the first-time manipulation. We observed brain activity in more regions during the first manipulation than during the other manipulations in the learning period, suggesting that controlling an object with an unfamiliar weight distribution involves a greater degree of sensorimotor interaction. As the number of manipulations increased, object-related manipulations were learned. Only M1, S1, and the vPMC were activated during the other three learned manipulations, indicating that motor control increasingly relies on memory with reduced sensorimotor interactions. Such learned manipulations were then transferred to a new grasp type in the transfer period. By comparing the brain activity in the first manipulation during the transfer period (T1) with that in the final learning period (L4), we determined that the PPC and SMA play roles in motor memory for transferring a learned manipulation.

As the weight distribution of the object was adjusted during the first grasp and lift manipulations, the properties of the object were uncertain. Thus, real-time sensorimotor interactions and adjustments in the force applied on the fingertip were needed to correct the roll of the object during the lifting motor. As predicted, we found that more brain areas were involved in the first manipulation. As proposed in previous reviews [19], the initial stage of performing an uncertain motor requires online sensory feedback from the fingertip to adjust the digit force. The adjustment in the forces represents an independent sensorimotor memory, which has also been shown to play a role in subsequent manipulations [34]. Our research examined brain activity associated with motor learning and memory formation. More brain regions were activated in the first manipulation of the learning period (L1), which might be considered key regions underlying sensorimotor interactions and generate motor memory. Initially, the unique involvement of the primary sensory cortex, including the ipsilateral S1 and bilateral primary visual cortex (V1), shown as the red parts in Figure 3A, was observed in L1, indicating high-level activity related to sensory input, which subsequently contributed to the integration of sensory and motor signals [38,39]. The bilateral V1 has been shown to play an essential role in visual working memory [40,41,42], and Zhao et al. emphasized the role of the ipsilateral V1 in visual working memory. In our research, we believe that the involvement of the ipsilateral S1 could be considered evidence that the primary sensory cortex plays a role in generating object-related memories. Interestingly, the bilateral MFG was found to be more activated during L1, which might provide additional evidence that the areas in the PFC connect the contralateral and ipsilateral S1 regions to achieve goal-directed recognition and memory [31,43,44]. Furthermore, the ipsilateral PPC, S1, V1, and V2 all showed larger activation during L1. The PPC is considered a high-level area that receives information from the primary sensory cortex and is responsible for generating predictions and correcting motor performance by generating a new motor control command, as well as other higher-order processes [32,33]. Taken together, the more activated areas involved in the first grasp and lift manipulation of an object with an unfamiliar weight distribution indicate a series of underlying functions. Our results suggest that areas in the ipsilateral S1, V1, PPC, and PFC are involved in sensorimotor interactions and the generation of motor-related memories.

As the number of manipulations increased, the areas activated in L2 to L4 and T2 to T4 highly overlapped, shown as the white parts in Figure 3A, with fewer regions activated than in L1, which reflects that the object manipulation has been learned. Our results reveal that the contralateral M1 and S1 regions and bilateral vPMC were responsible for motor control during the learned manipulation. The involvement of M1 supported its functions of storing and retrieving sensorimotor memories of grasp forces [45,46,47,48]. However, several studies [49,50] have suggested that sensorimotor interactions still affect motor control during skilled object manipulation. In our experiments, the sustained activation in S1 and M1 reflected that sensorimotor interactions are still being processed when performing a learned manipulation. So, we believe that both the online feedback of the digit position and force and memories work together. However, the reduced involvement of high-level areas while performing the learned manipulation suggests a decline in sensorimotor processing demands, which may be attributed to the role of memory in the execution of learned manipulations.

In addition to the vPMC, S1, and M1 regions involved in the motor memory of a learned manipulation, we identified other brain areas responsible for motor memory during the transfer of a learned manipulation to a new manipulation type. As the two-digit grasp [5,6,7] and three-digit grasp [8] motors require different fingertip forces and hand muscle forces, the transfer period when adding or removing a digit involved in the manipulation depends on a new motor plan. In our research, by comparing the results during the first manipulation of the transfer period (T1) with those during the fourth manipulation of the learning period (L4), we identified the contralateral SMG, PoCG, and ipsilateral SFG as possibly being responsible for the new type of motor plan and control shown in Figure 4. The characteristic areas in the SFG are also part of the SMA, which has been reported to be highly involved in motor planning during voluntary movement [51,52] and other goal-directed motors [53,54]. Considering that the motor goal, grasping and lifting the T-object, remained identical during L4 and T1, we suggest that the ipsilateral SMA’s role in goal-directed motor control might specifically relate to the application of forces on the fingertips. Additionally, we identified the contralateral PoCG and SMG in the PPC as being involved in the new type of motor planning. The PPC has been shown to be involved in the coordination of fingertip forces [55]. Interestingly, another study [56] suggested that PPC is not only involved in motor memory but also in force and position perception. Given that the sensory input was different when adding or removing a digit to grasp in T1, along with the high-level real-time sensorimotor interactions and adjustments in the force in L1, we suggest that the PPC might be responsible for processing the real-time sensory input with the learned motor memory. Our findings improve our understanding of the role of the PPC in motor memory, highlighting how it integrates sensory information from memory with real-time input to generate novel motor control signals that guide the precise reapplication of fingertip forces.

In our study, only male participants were recruited to complete the fMRI experiment due to their generally better stamina and hand muscle strength. However, as gender differences might offer alternative insights into motor learning mechanisms, future research might investigate female participants’ motor learning and transfer processes using lighter masses or shorter experimental durations by decreasing the trial numbers. Additionally, the understanding of the role of SMA and PPC in motor learning and transferring might provide new perspectives on understanding neurodegeneration diseases and developing several treatments. For instance, recent studies have reported changes in the cortical thinning of PFC and PPC of amyotrophic lateral sclerosis (ALS) patients from a structural perspective [57], as well as decreased connectivity between the right middle frontal gyrus and the PPC of ALS patients compared with the connectivity of healthy controls from a functional perspective [58]. Based on these findings, we suggest investigating the brain functions underlying motor transfer using the same grasping and lifting task in ALS patients. Exploring motor transfer mechanisms in ALS patients and comparing these findings with our proposed activations could deepen our understanding of ALS and develop therapeutic approaches.

## 5. Conclusions

In conclusion, our results suggest the brain mechanisms involved in transferring learned manipulation skills to new types of manipulations. Our results demonstrate that manipulating an object with an unfamiliar weight distribution involves more brain regions during the initial grasp-and-lift task. As the manipulation is learned, motor control increasingly relies on memories from the M1, S1, and vPMC regions, resulting in the activation of fewer brain regions. Additionally, during the transfer to a new manipulation type, the involvement of the PPC and SMA suggests their critical roles in planning and executing new motor strategies from learned manipulations. This highlights the brain’s adaptability in transferring learned dexterous object manipulations.

## Data Availability

The original contributions presented in the study are included in the article, further inquiries can be directed to the corresponding author.
